# Temperature and Gate-Length Dependence of Subthreshold RF Detection in GaN HEMTs

**DOI:** 10.3390/s22041515

**Published:** 2022-02-15

**Authors:** Gaudencio Paz-Martínez, Ignacio Íñiguez-de-la-Torre, Héctor Sánchez-Martín, José Antonio Novoa-López, Virginie Hoel, Yvon Cordier, Javier Mateos, Tomás González

**Affiliations:** 1 Departamento de Física Aplicada and USAL-NANOLAB, Universidad de Salamanca, E-37008 Salamanca, Spain; indy@usal.es (I.Í.-d.-l.-T.); hectorsanchezmartin@usal.es (H.S.-M.); joseantonionolo@usal.es (J.A.N.-L.); javierm@usal.es (J.M.); tomasg@usal.es (T.G.); 2 UMR 8520—IEMN—Institut d’Electronique de Microélectronique et de Nanotechnologie, CNRS, Centrale Lille, Université Polytechnique Hauts-de-France, Université de Lille, F-59000 Lille, France; virginie.hoel@univ-lille.fr; 3 Université Côte d’Azur, CNRS, CRHEA, rue Bernard Grégory, F-06560 Valbonne, France; yvon.cordier@crhea.cnrs.fr

**Keywords:** GaN HEMTs, RF detectors, bulk and surface traps, gate leakage, responsivity, third-quadrant conduction

## Abstract

The responsivity of AlGaN/GaN high-electron mobility transistors (HEMTs) when operating as zero-bias RF detectors in the subthreshold regime exhibits different behaviors depending on the operating temperature and gate length of the transistors. We have characterized in temperature (8–400 K) the detection performance of HEMTs with different gate lengths (75–250 nm). The detection results at 1 GHz can be reproduced by a quasi-static model, which allows us to interpret them by inspection of the output $I_{D}\;$−$\;V_{DS}$ curves of the transistors. We explain the different behaviors observed in terms of the presence or absence of a shift in the zero-current operating point originating from the existence of the gate-leakage current jointly with temperature effects related to the ionization of bulk traps.

## 1. Introduction

Field-effect transistors (FETs), thanks to their intrinsic nonlinearities, exhibit competitive performance as detectors of RF and THz signals [1]. Different FET technologies have been explored to this end: Si CMOS, graphene FETs, HEMTs based on GaN, GaAs, InGaAs, InAs, etc. [1,2,3,4,5,6,7,8,9,10,11]. Both when operating as zero-current and zero-voltage detectors (ZCDs and ZVDs, respectively), the maximum value of responsivity β is typically achieved around threshold conditions. However, when entering the subthreshold region (the focus of this study), in ZVDs β always vanishes, whereas in ZCDs, different behaviors can be observed. In some studies, a decrease in β similar to that of ZVDs takes place [1,11], whereas in others, a saturation at the maximum value achieved around the threshold voltage is observed [6,7,10]. Attempts to explain these behaviors as due to the influence of gate-leakage current [4] or detector loading conditions [2,5] have been reported, but a comprehensive physical explanation is still lacking. In this study, we tackle this problem in the particular case of GaN HEMTs [4,5,6,7,8,9]. These devices present issues related to traps and leakage currents, widely studied and well understood when working as amplifiers [12,13,14]. Here, we focus on their influence on subthreshold zero-bias RF detection, with the devices working under totally different conditions. GaN HEMTs with several different gate lengths are measured in a wide temperature range, finding the aforementioned diverse behaviors of β in the subthreshold results. We provide a global interpretation for them in terms of the competition between buffer and gate leakage currents, ionization of traps and third-quadrant conduction. Even if the results reported here are specific to GaN HEMTs, a similar influence of the gate-length and associated short-channel effects could be expected in other technologies. However, we have to remark that our conclusions are only valid up to frequencies for which the resistive mixing theory for current rectification is applicable. Within this theory, the RF responsivity can be related to the non-linearity of the *I* − *V* curves of the devices, and were applied in this study through a quasi-static analytical model. The same rectification mechanism still holds above the cut-off frequency of the devices as long as it is the result of distributed mixing, when the channel of the FET can no longer be treated as a lumped element, but rather as an ultra-high-frequency waveguide [15,16]. This is the frequency range typically addressed in the literature of FET-based detectors, where our conclusions can be directly applicable. However, it is not the case when entering into the THz range, where thermoelectric or plasmonic effects are involved in the rectification [17,18], and thus are not directly related to the DC characteristics of the devices.

## 2. Devices and Methods

Two-finger AlGaN/GaN HEMTs grown on Si substrate with gate lengths $L_{g}$ = 75, 150 and 250 nm, drain-to-source distance $L_{DS}$ = 2.5 µm and width 2 × 25 µm are analyzed [19,20,21]. The epilayer consists of a Si substrate with a 1.73 μm thick GaN buffer, a 1 nm AlN spacer, 14 nm of AlGaN (29% Al) and a top 0.5 nm GaN cap layer (more information about the devices is provided in Refs. [15,16,17]). In order to obtain a high-resistivity buffer and improve the device pinch-off, the n-type conductivity of the GaN buffer must be compensated with p-type doping. Typically, intentional (or unintentional) Fe or C doping is used to this end [22,23,24,25]. In our devices, C atoms in a concentration about 10^17^ cm^−^^3^ play this role [26]. This will have a strong influence on the results obtained, mainly at low temperature, when the acceptor ions are not ionized and the pinch-off behavior of the GaN HEMTs is degraded.

For the measurements, the sample, placed inside a LakeShore CRX-VF (Lake Shore Cryotronics, Westerville, OH, USA) cryogenic probe station, was connected on-wafer to a Keysight N5244A PNA-X VNA, used as RF source, and a two-channel Keysight B2902A SMU, Keysight Technologies, Santa Rosa, CA, USA, (with 15 GΩ input impedance), which enabled both biasing of the device and recording the DC output. The injected RF signal of power $P_{in}$ was coupled to the drain terminal and the average DC voltage shift $∆V_{DS}$, whereas biasing with $I_{D}$ = 0 (or the DC current variation $∆I_{D}$, while biasing with $V_{DS}=0$), was taken as the output signal; thus, the device operated as ZCD (or ZVD). The resultant responsivities are calculated as $\beta_{ZCD}=∆V_{DS}/P_{in}$ or $\beta_{ZVD}=∆I_{D}/P_{in}$, and the corresponding noise equivalent powers as $NEP_{ZCD}={\left(4k_{B}TR\right)}^{1/2}/\beta_{ZCD}$ and $NEP_{ZVD}={\left(4k_{B}T/R\right)}^{1/2}/\beta_{ZVD}$, with the resistance *R* measured at the zero-current or zero-voltage operating point, respectively. Operating temperatures *T* of 8, 20, 100, 200, 300 and 400 K were explored. The frequency of the RF signal was 1 GHz and $P_{in}$ = −15 dBm. We remark that a simple quasi-static model based only on the DC $I_{D}-V_{DS}$ curves [27] accurately reproduces the measurements of the RF responsivity in the entire gate-bias sweep and for all the temperature range.

## 3. Results and Discussion

**Figure 1:**
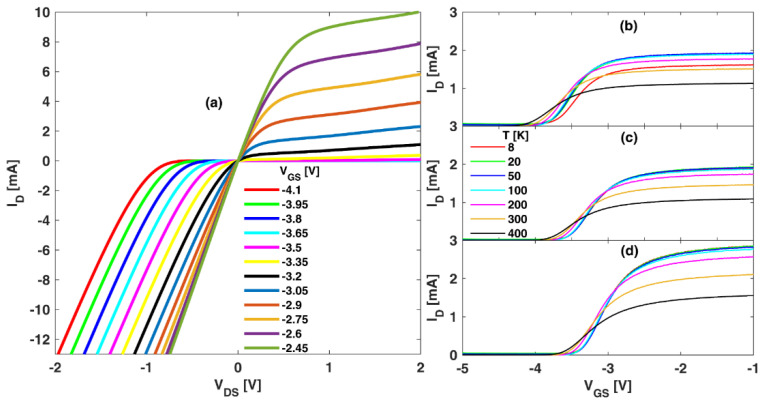
(**a**) $I_{D}\;$−$\;V_{DS}$ output characteristics of the HEMT with $L_{g}$ = 250 nm and T = 300 K. All the operation regions are shown, including the third-quadrant conduction for negative *V_DS_* values. $I_{D}\;$−$\;V_{GS}$ transfer characteristics for *V_DS_* = 0.1 V from 8 to 400 K for the three devices under test: (**b**) *L_g_* = 75 nm, (**c**) *L_g_* = 150 nm and (**d**) *L_g_* = 250 nm.

Figure 1 shows examples of the output and transfer I − V curves of our HEMTs. The output characteristics of Figure 1a, only presented for the case $L_{g}$ = 250 nm and *T* = 300 K, show the good transistor behavior displayed by all the devices at every T (all of them presented later). A significant increase in *I_D_* is clearly visible for negative $V_{DS}$ when *V_GD_* = *V_GS_* − *V_DS_* > *V_th_*, i.e., $V_{DS}<V_{GS}-V_{th}$ (with $V_{th}$ the threshold voltage of the transistors), which corresponds to the so-called “third-quadrant conduction” of FETs [9,28,29]. As explained later, this effect will be of importance for the position of the zero-*I_D_* bias point in some cases (when the buffer contribution to the drain leakage current is nearly zero and third-quadrant conduction is needed to compensate the gate contribution). The transfer characteristics in Figure 1b,c show a slight shift of $V_{th}$ to more negative values the higher the T and the shorter the $L_{g}$.

**Figure 2:**
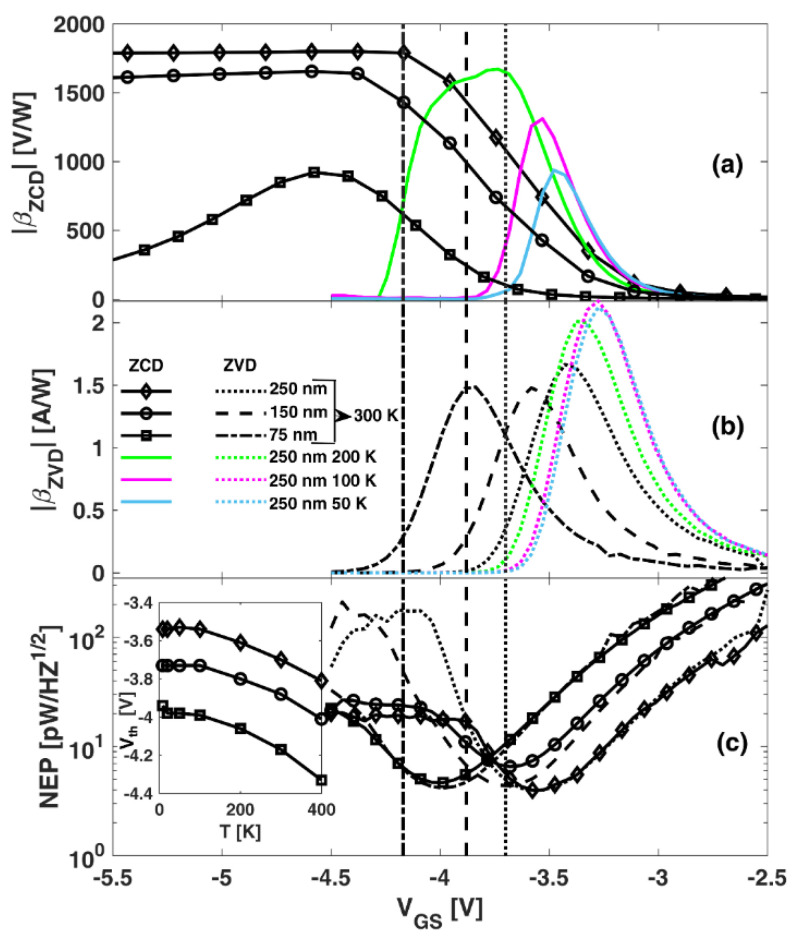
(**a**) ZCD and (**b**) ZVD responsivities as a function of $V_{GS}$ for transistors with different $L_{G}$ values measured at several *T*. (**c**) Corresponding *NEP*s at 300 K. The inset in (**c**) shows $V_{th}$ of the transistors as a function of *T*, determined at $V_{DS}$ = 0.1 V. The vertical lines indicate the value of $V_{th}$ for the different gate lengths at 300 K.

Figure 2 shows (a) $\beta_{ZCD}$ and (b) $\beta_{ZVD}$ measured in transistors with different $L_{g}$ values operating at several values of *T*. The inset in Figure 2c shows the dependence of the threshold voltage of the transistors *V_th_* on $L_{g}$ and *T*. Both responsivities take very low values in open-channel conditions and increase when $V_{GS}$ approaches $V_{th}$. However, when entering the subthreshold region, whereas $\beta_{ZVD}$ decreases (after reaching a maximum around $V_{th}$) irrespectively of the values of $L_{g}$ and *T*, $\beta_{ZCD}$ tends to keep its maximum value for a wider range of *V_GS_* as $L_{g}$ becomes longer and *T* increases above 100 K. Remarkably, in the case of *L_g_* = 250 nm and *T* ≥ 300 K, $\beta_{ZCD}$ remains constant in the whole measured range of subthreshold operation, as in Reference [6]. In general, increasing values of *L_g_* and *T* (below 300 K) lead to higher values of $\beta_{ZCD}$. However, when *T* exceeds 300 K, $\beta_{ZCD}$ decreases [6].

The sensitivity of the transistors as RF detectors is assessed in terms of the NEP. *NEP_ZCD_* and $NEP_{ZVD}$, shown in Figure 2c at 300 K, exhibited the typical minimum at *V_GS_*, slightly higher than $V_{th}$ [2,6,8]. Interestingly, thanks to the plateau found in $\beta_{ZCD}$ when entering in the subthreshold regime, for *L_g_* = 150 and 250 nm, the ZCD scheme provides improved values of NEP as compared with the ZVD case.

We have confirmed (results no shown here) that if the load resistance in ZCD measurements is reduced, the responsivity is attenuated in subthreshold conditions, such as in [2,5], because the HEMT, operating in pinch-off, is not able to deliver the increasing current required by a lower load resistance to maintain the same responsivity. On the other hand, the observed dependencies are essentially the same when the frequency of the RF signal is increased up to 43.5 GHz (limit of our setup).

A quasi-static (QS) model [27] based on the second-order Taylor series expansion of the current as a function of the voltage is able to reproduce the previous results at 1 GHz. The parameters of the model are calculated from the measured static $I_{D}\;-\;V_{DS}$ characteristics. The unmatched current and voltage responsivities provided by the model for ZVD ($\beta_{QSI}$) and ZCD ($\beta_{QSV}$) are given by:

(1) $$\beta_{QSI}=\frac{1}{2}\gamma \left(1-{\left|\Gamma \right|}^{2}\right),\;\mathrm{in}\;A/W\;\mathrm{units},$$

(2) $$\beta_{QSV}=-\frac{1}{2}R_{DS}\gamma \left(1-{\left|\Gamma \right|}^{2}\right),\;\mathrm{in}\;V/W\;\mathrm{units},$$

where *R_DS_* = (*dI*/*dV*)^−1^ is the channel resistance and *γ* is the bowing coefficient, a measure of the nonlinearity of the I − V curve, given by

(3) $$\gamma =\left(\frac{\partial^{2}I}{\partial V^{2}}\right)/\left(\frac{\partial I}{\partial V}\right)\;\mathrm{in}\;V^{-1}\;\mathrm{units}.$$

**Figure 3:**
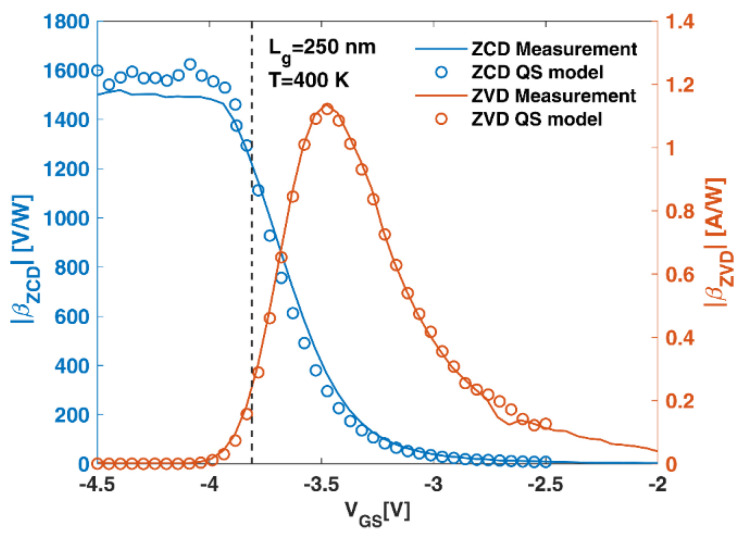
Measurements of the responsivity (lines) in ZCD and ZVD at *T* = 400 K for the device with $L_{g}$ = 250 nm compared with the values provided by the quasi-static model (symbols). The dotted vertical line represents the threshold voltage.

Finally, Γ = (*Z_d_* − *Z*_0_)/(*Z_d_* + *Z*_0_) is the reflection coefficient, with *Z_d_* as the device impedance and *Z*_0_ as the output impedance of the RF source, being 50 Ω. Figure 3 shows, as an example, the values of the responsivity measured at *T* = 400 K for the device with $L_{g}\;$= 250 nm (lines) and those calculated with the QS model (symbols). The vertical dotted line represents the threshold voltage (−3.81 V). As observed, in sub-threshold operation, the (positive) ZVD responsivity tends to zero, whereas the (negative) ZCD responsivity tends to a constant value, the model fitting very well to the measurements in both cases.

**Figure 4:**
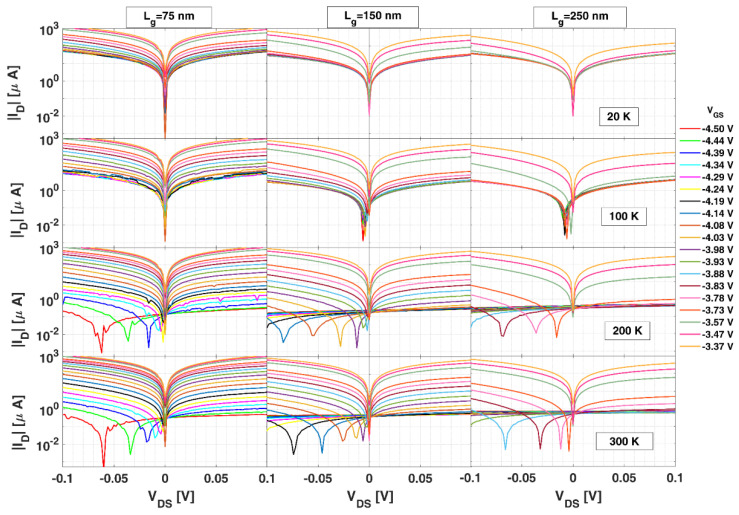
|$I_{D}$| vs. $V_{DS}$ for several values of $V_{GS}$ in transistors with different $L_{g}$ measured at several *T*. The minimum in the curves corresponds to the value of *V_DS_* at which the current changes sign.

To provide a physical explanation for the results shown in Figure 2, and because they can be reproduced with the quasi-static model, in Figure 4 we present the DC output characteristics around the origin. They are represented in log-scale in order to better observe the behavior of *I_D_* when approaching its zero value in the $V_{DS}$ range where the ZCD ($I_{D}\;$= 0) and ZVD ($V_{DS}$ = 0) operating points are expected to be. Below 200 K, the transistors exhibit a poor pinch-off; both operating points are essentially at the origin, and the curves become slightly asymmetric around *V_DS_* = 0 when $V_{GS}$ approaches $V_{th}$, leading to a more pronounced nonlinearity and a maximum in the responsivities. For higher *T*, an improved pinch-off is observed. This comes with a shift of the zero-$I_{D}$ operating point to negative values of $V_{DS}$ in subthreshold conditions, around which the asymmetry of the curves is stronger, leading to an enhanced responsivity. These effects, at the origin of the behavior found in $\beta_{ZCD}$, are more pronounced the longer the $L_{g}$.

**Figure 5:**
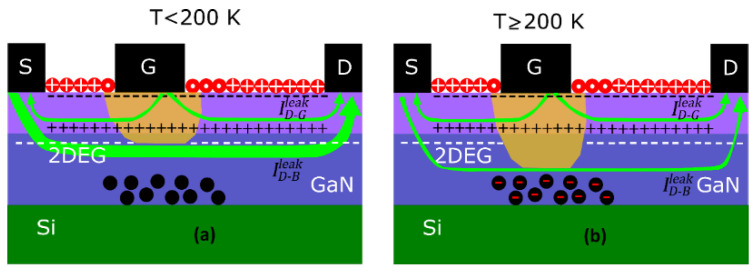
Scheme of fixed charges and electron fluxes in the subthreshold regime at (**a**) low and (**b**) high *T.* The case shown corresponds to $V_{DS}$ > 0. In cases where $V_{DS}$ < 0, the flow of electrons through the buffer would change direction, whereas those injected by the gate remain unaltered.

**Figure 6:**
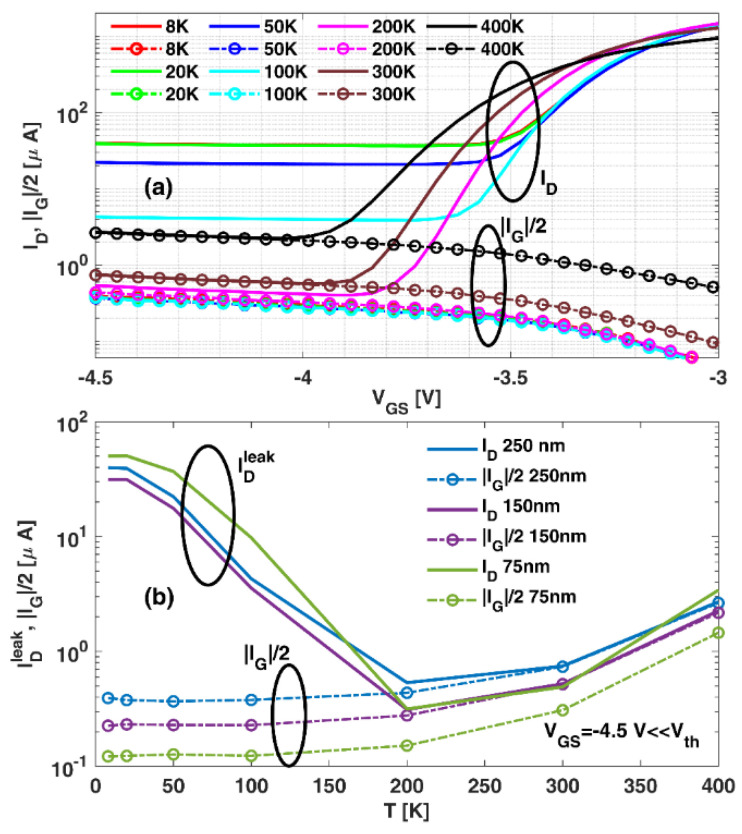
$I_{D}^{leak}$ and |*I_G_*|/2 at $V_{DS}$ = 0.1 V (**a**) as a function of $V_{GS}$ measured at different *T* for the case of the transistor with *L_g_* = 250 nm and (**b**) as a function of *T* for $V_{GS}$= −4.5 V for the three transistors under test.

**Figure 7:**
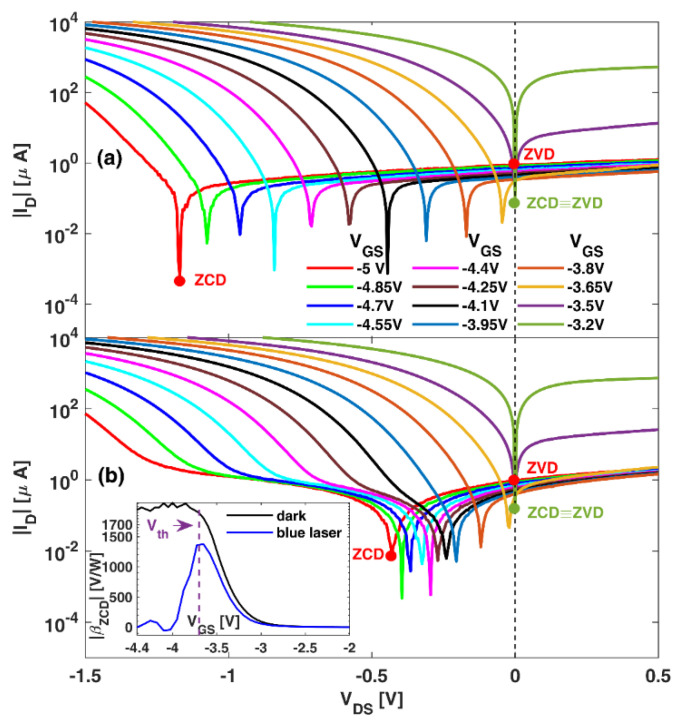
(**a**) *|*$I_{D}$*|* vs. $V_{DS}$ for several values of $V_{GS}$ measured at 300 K for $L_{g}$ = 250 nm. ZCD and ZVD operating points are marked in green and red for $V_{GS}$ values below $V_{th}$ (−3.70 V) and deep in the subthreshold, respectively. (**b**) *|*$I_{D}$*|* vs. $V_{DS}$ for several values of $V_{GS}$ under blue laser illumination measured at 300 K. The inset shows the ZCD responsivity in the dark and under blue-laser illumination.

The shift in the zero-$I_{D}$ operating point is caused by the leakage currents present in the transistors. The two main contributions to the drain-leakage current $I_{D}^{leak}$ in the subthreshold regime are schematically depicted in Figure 5. A primary contribution, $I_{D-G}^{leak}$, comes from the electrons tunneled through the gate which, due to the practically symmetric electric potential profile present at low $V_{DS}$ under the gate, move towards its nearest edge. Thus, the gate current $I_{G}$ is split into two halves directed towards the source and drain [30,31]; thus, $I_{D-G}^{leak}≅\;\left|I_{G}\right|/2$, with $I_{G}$ as the gate current. The second contribution is the source-to-drain buffer leakage $I_{D-B}^{leak}$ due to a poor electron confinement in the channel [32]. Figure 6a shows the transfer characteristics of the transistor with $L_{g}$ = 250 nm at $V_{DS}\;$ = 0.1 V and different *T*, jointly with $I_{D-G}^{leak}$. As observed in Figure 6b, for all the three gate lengths, a different behavior is observed for a *T* lower or higher than 200 K depending on the level of ionization of the acceptor-like doping present in the buffer:

- At *T* < 200 K, the low ionization level of the GaN buffer acceptor levels allows for a significant leakage of drain current through the buffer $I_{D-B}^{leak}$ (see Figure 5a), which becomes the main contribution to $I_{D}^{leak}$ and the zero-$I_{D}$ point remains close to $V_{DS}=0$ (see Figure 4 for 20 K and 100 K);
- At *T* > 200 K, when full ionization is achieved, $I_{D-B}^{leak}$ is strongly suppressed, leading to an improved pinch-off (see Figure 4 for 200 and 300 K) [32,33,34,35,36]. Consequently, the two drain-leakage contributions become of the same order (µA), as sketched in Figure 5b, or even $I_{D-G}^{leak}$ as key:
  ○ For *L_g_* = 75 nm, even if $I_{D-B}^{leak}$ is reduced, it is still significant (due to short channel effects) and of the order of $I_{D-G}^{leak}$, as can be deduced from Figure 6b, where it is observed that $I_{D}^{leak}$ > $\left|I_{G}\right|/2$. In such a case, because $I_{D-G}^{leak}$ is positive and almost constant with $V_{DS}$, the zero-$I_{D}$ condition is accomplished for a negative value of $V_{DS}$, for which a negative $I_{D-B}^{leak}$ compensates $I_{D-G}^{leak}$. As $V_{GS}$ takes higher negative values, $I_{D-B}^{leak}$ decreases and a higher negative value of $V_{DS}$ is necessary to achieve zero current, but not yet reaching the third-quadrant conduction condition. At such a zero-$I_{D}$ point, the asymmetry of the $I_{D}-V_{DS}$ curves is degraded due to the symmetric contribution of $I_{D-B}^{leak}$ (similarly to what happens at *T* < 200 K, regardless of the gate length). This is the reason for the decrease in $\beta_{ZCD}$ observed at 300 K for this gate length in subthreshold conditions (see Figure 2a);
  ○ For long gates $(L_{g}$ = 150 and 250 nm), thanks to the better gate control of the channel concentration (short channel effects are absent in this case), $I_{D-B}^{leak}$ is further reduced and $I_{D}^{leak}$ essentially coincides with $\left|I_{G}\right|/2$ (Figure 6b). Thus, the zero-$I_{D}$ point is only achieved when $V_{DS}$ reaches a value near $V_{GS}\;-$ $V_{th}$, at which the onset of third-quadrant conduction takes place in the transistor [9,28,29], and the significant increase in $I_{D}$ (no longer due to buffer leakage but to the opening of the channel for sufficiently negative values of *V_DS_*) can compensate $I_{D-G}^{leak}$. These are the conditions shown in Figure 7a, for $L_{g}$ = 250 nm and *T* = 300 K, for which the ZCD operation takes place at a point ($I_{D}$ = 0) with strong asymmetry (mainly due to the pronounced increase in $I_{D}$ once third-quadrant conduction is reached). Consequently, the high value of $\beta_{ZCD}$ remains almost constant for $V_{GS}\;$< $V_{th}$.

In contrast to the complex behavior of the zero-$I_{D}$ point when entering into the subthreshold regime, the $I_{D}$ − $V_{DS}$ curve is essentially linear at the zero-$V_{DS}$ point for all temperatures and gate lengths. This originates in the decreasing values of $\beta_{ZVD}$ when $V_{GS}$ approaches $V_{th}$, as observed in Figure 3.

Interestingly, when the device is illuminated with a 405 nm laser diode, the case shown in Figure 7b, the zero-$I_{D}$ conditions are achieved at lower values of $V_{DS}$, where asymmetry is smaller. Consequently, $\beta_{ZCD}$ decreases in the subthreshold (blue curve in the inset) and exhibits a maximum as in low temperatures (*T* ≤ 100 K). We attribute this behavior to the ionization of deep donor-like traps, most probably located at the surface of the device in the vicinity of the gate, such as those originating in virtual-gate effects [37,38,39], although they could also be located in the buffer [36]. These traps, by becoming positively charged when electrons are released, weaken the pinch-off of the channel, allowing the presence of a higher $I_{D-B}^{leak}$; thus, $\beta_{ZCD}$ shows a similar behavior to that observed at low *T*. Figure 7b clearly shows the onset of the third-quadrant conduction, evidenced by a sharp increase in $I_{D}$ for $V_{DS}$ < $V_{GS}-V_{th}$, whereas above that value the slope of the $I_{D}\;-\;V_{DS}$ is small (and nearly constant). The key point here is that the illumination is able to shift the location of the zero-$I_{D}$ bias point from the onset of the third-quadrant conduction (as occurs in dark conditions) to the almost flat $I_{D}-V_{DS}$ region, thus leading to the decrease in $\beta_{ZCD}$.

## 4. Conclusions

The different behaviors exhibited by the subthreshold responsivity of zero-bias RF detectors based on AlGaN/GaN HEMTs (with the signal coupled to the drain) have been explained in terms of the competition between the two (gate and buffer) contributions to the drain leakage current and the influence of bulk traps. When the channel is properly pinched off (high temperature and long gate), due to the presence of a gate-leakage current, the zero-$I_{D}$ point is shifted to negative values of $V_{DS}$, where the nonlinearity of the $I_{D}-V_{DS}$ curves is enhanced, leading to the saturation of the responsivity at its maximum value in subthreshold operation and thus to an improved sensitivity in terms of the $NEP_{ZCD}$. The shift is not present (or is less pronounced) at low temperature and/or in transistors with strong short-channel effects, cases in which the responsivity just shows a maximum around the threshold voltage for then decreasing when entering deep into the subthreshold region (leading to a poorer *NEP*), as happens in all cases when detection takes place under zero-$V_{DS}$ operation. While the trap-related temperature effects are distinctive of GaN HEMTs, the influence of gate length and associated short-channel effects discussed in this paper could also be of importance in other FET technologies.

Finally, we remark that although in this study we used a drain-coupling scheme for the RF characterization of the devices under probes, in the case of free-space THz characterization, a gate-coupling scheme is used more often [3,6,8,10,11]. However, the conclusions drawn from our experiments are also valid for the case of gate coupling, because the responsivity for both schemes is related to the non-linearity of the *I* − *V* curves of the HEMTs (around the DC bias point).

## Data Availability

The data that support the findings of this study are available from the corresponding author upon reasonable request.
